# Development for Probiotics Based Insulin Delivery System

**DOI:** 10.3390/cimb47030137

**Published:** 2025-02-21

**Authors:** Byung Chull An, Jusung Lee, Hye Yeon Won, Yongku Ryu, Myung Jun Chung

**Affiliations:** R&D Center, Cell Biotech, Co., Ltd., 50, Aegibong-ro 409 beon-gil, Gaegok-ri, Wolgot-myeon, Gimpo-si 10003, Gyeonggi-do, Republic of Korea; bcan@cellbiotech.com (B.C.A.); jslee@cellbiotech.com (J.L.); hywon@cellbiotech.com (H.Y.W.); ykryu@cellbiotech.com (Y.R.)

**Keywords:** diabetes mellitus (DM), single-chain insulin (SCI), pancreatic beta-cell proliferation, adipocytes differentiation, glucose uptake

## Abstract

Probiotics show beneficial effects on diabetes mellitus (DM). If probiotics can secrete the recombinant insulins that may help suppress DM development, then it would likely have very few adverse side effects. To produce insulin analogs in bacteria, recombinant insulin (insulin-CBT1) should be the single-chain insulin (SCI) similar to proinsulin. However, insulin-CBT1 should allow the protein to activate insulin receptors directly without the need for proteolytic cleavage. In this study, we evaluated the effect of the flexible linker peptide on the physical and structural characteristics of insulin-CBT1 compared with commercial insulin (c-insulin). In the results, the linker peptide had marked effects on polarity and structure by increasing the α-helix content (19.3%→25.6%). Furthermore, insulin-CBT1 induced MIN6 proliferation 1.75-fold more than c-insulin, whereas differentiation and glucose uptake rates by 3T3-L1 were 39% and 15% lower, respectively. The biological anti-diabetes properties of insulin-CBT1 were well evaluated compared with c-insulin. Furthermore, we first suggest a special method for oral administration of insulin-CBT 1 without damage to the digestive tract. We developed an insulin-CBT1 delivery system using *Pediococcus pentosaceus* (PP), which has been reported as a potential bacteria in DM. First, insulin-CBT1 was harbored in pCBT2-24, which verified the expression and secretion vector system of PP. We finally confirmed that PP-insulin-CBT1 successfully secreted insulin-CBT1 proteins to culture media. These results presented herein open up new avenues to developing therapeutic options for DM.

## 1. Introduction

A relative deficiency of insulin or a lack of sensitivity to endogenous insulin reduces glucose tolerance and can cause diabetes mellitus (DM).

Lack of insulin production or sensitivity leads to hyperglycemia. Long-term exposure to high glucose concentrations is associated with a marked increase in the incidence of complications such as vascular disease, coronary heart disease, retinopathy, nephropathy, and neuropathy. According to MFD (the International Diabetes Federation) estimates, in 2019, approximately 463 million adults aged 20–79 years were living with DM; moreover, this will rise to 700 million by 2045 [https://www.idf.org/, accessed on 21 August 2023]. Unfortunately, DM causes 4.2 million deaths per year [https://www.idf.org/]. Recombinant human (rh)-insulin, a therapeutic analog of endogenous insulin, was developed to treat DM. Insulin, a 51-residue (about 6 kDa) anabolic protein hormone secreted by β-cells in the Islets of Langerhans in the pancreas, plays a central role in regulating glucose levels in plasma by binding to cell-surface insulin receptors (INSRs) [1]. Mature insulin, comprising two chains (A and B) connected by disulfide bonds, is generated by post-translational cleavage of a single-chain precursor called proinsulin [1]. Insulin replacement therapy is the standard treatment for both type 1 and advanced type 2 DM [2]. Biosynthetic rh-insulin generated using recombinant DNA technology enables large-scale production in microorganisms [*Escherichia coli* (*E. coli*) and yeasts]. Therefore, rh-insulin can be provided worldwide at affordable prices. Although patients can switch safely and effectively from animal-derived semisynthetic human insulin to rh-insulin with no change in the expected dosage [3], global data suggest that the incidence of insulin-dependent DM may reach as high as ~9.5% [4] and that demand for insulin can be as high as 0.5–1 g/patient/year [5]. Therefore, rh-insulin will require higher productivity.

To overcome this, various strategies have been proposed to improve the production of rh-insulin. These include production in bacteria. Indeed, studies suggest that h-proinsulin can be expressed successfully in *E. coli* strains, followed by the production of mature rh-insulin via refolding reactions, precipitation, purification, and enzymatic cleavage (with trypsin and carboxypeptidase B) [5,6,7]. Other studies expressed h-insulin A and B chains in two different *E. coli* cells and then mixed the purified insulin A and B chains, followed by reduction–reoxidation to ensure the correct joining of the disulfide bonds [8,9]. However, this form of rh-insulin shows low activity in humans [10]. Other approaches include increasing efficacy. To develop rh-insulin with short-acting or long-acting activity, many studies used protein engineering technology. Short-acting rh-insulin can be injected immediately before meals because it acts much more quickly than native rh-insulin [11,12]. Homo-oligomerization (dimers and hexamers) of native h-insulin can slow absorption [13,14]; therefore, to improve the absorption of insulin analogs, their ability to self-associate can be reduced below that of native insulin [15,16]. Another approach, long-acting rh-insulin, involves genetic engineering of h-insulin to alter its absorption and action time in the body. Altering amino acids to confer a positive net charge makes analogs less soluble, thereby delaying absorption [17,18,19]. Insulin analogs increase our understanding of the structure-function relationships and associated biochemical mechanisms underlying INSR binding [20]. Although alternative approaches to the synthesis of recombinant r-proinsulin have been developed, this molecule requires post-translational modification to exert biological activity [21,22,23]. Because the affinity of proinsulin for the INSR is only 1–2% that of mature insulin [24], alternative strategies for commercial production of single-chain insulin (SCI) analogs [which do not require post-translational modification (PTM) to exert their biological activity] are being developed [25]. Here, we used protein engineering to improve the function of an SCI analog (insulin-CBT1). We replaced the C-peptide with a flexible linker peptide that allows flexible folding and disulfide bond pairing between the A and B chains; because this new folding pattern does not interfere with binding to the INSR, this molecule does not require cleavage for its activity. Next, we examined the structure/function relationships of insulin-CBT1 and its interaction with the INSR on the surface of pancreatic (MIN6) and pre-adipocyte (3T3-L1) cells. Furthermore, we examined insulin-CBT1-mediated specific signal cascades in MIN6 and 3T3-L1 cells.

In recent years, probiotics have been seriously considered as the bacterial carrier for drug delivery systems (DDSs) and have been proven to be effective therapeutic agents in the intestine. Probiotics are, by definition, safe to ingest, and when consumed in sufficient amounts, they confer diverse health benefits [26]. In addition, several studies have demonstrated that probiotics have a positive impact on host health; the beneficial effects of probiotics are strain-specific and related to host physiology [27]. Recently, *Pediococcus pentosaceus* has been reported as a potential bacteria that could be helpful to DM, and *P. pentosaceus* has α-amylase and α-glucosidase inhibitory activity [28]. We have previously developed a recombinant *P. pentosaceus* SL4 (PP) DDS-harboring protein drug for oral administration [29]. If PP can secrete the recombinant insulin-CBT1 proteins, that may help suppress DM development; furthermore, it would likely supply the original benefits of probiotics. To produce insulin analogs in bacteria, recombinant insulin (insulin-CBT1) should be harbored in pCBT2-24, which has verified the expression and secretion vector system for PP [29]. We finally confirmed that insulin-CBT1 was successfully expressed and then secreted to culture media. In a further study, transgenic PP (pCBT2-24-insulin-CBT1) will be tested, which may help suppress DM development in DM animal models by oral administration.

## 2. Materials and Methods

### 2.1. Cell Lines and Culture Conditions

*Escherichia coli* (*E. coli*) cells were cultured for 18–24 h at 37 °C in LB broth (Difco, Detroit, MI, USA). Mouse-derived pancreatic cells (MIN6) and pre-adipocyte cells (3T3-L1) were purchased from the American Type Culture Collection and maintained under 5% CO_2_/37 °C in Roswell Park Memorial Institute (RPMI)-1640 medium (Gibco, Grand Island, NY, USA) or Dulbecco’s Modified Eagle Medium (DMEM), respectively, containing 10% fetal bovine serum (FBS, Gibco) and 1% penicillin/streptomycin (Gibco). Adipocyte differentiation of 3T3-L1 cells was performed using the modified protocol of Lane et al. [30].

### 2.2. Construction of the Plasmid Harboring Insulin-CBT1 for Expression in E. coli

**Figure 1:**
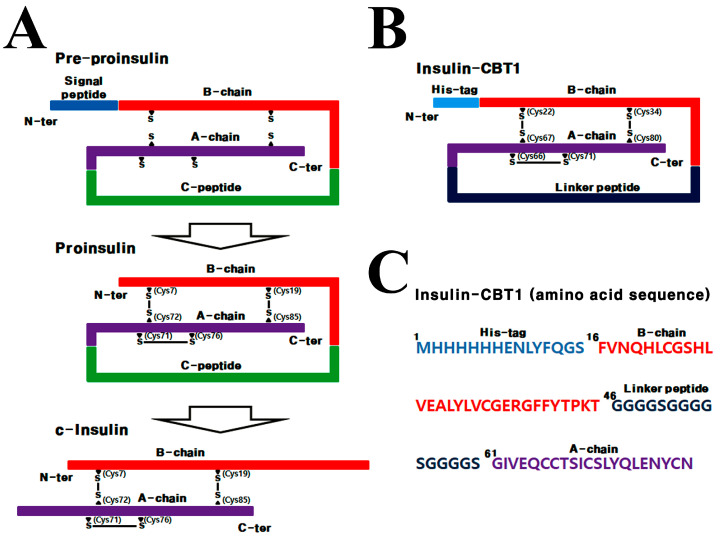
Schematic illustration of the insulin analogs. (**A**) Post-translational modification of c-insulin. (**B**) Schematic illustration of insulin-CBT1. (**C**) Each motif was indicated on the amino acid sequence of insulin-CBT1.

Figure 1 shows a schematic representation of the expression vector pET22b(+) (Novagen, Cambridge, MA, USA) and the expression constructs comprising a 6×His-tagged B chain ligated to a flexible linker peptide-fused A chain of the h-insulin gene. The ATUM-codon optimized DNA fragment of insulin-CBT1 (ATUM. bio, Newark, CA, USA) is flanked by NcoI and SalI restriction sites. The insulin-CBT1 sequences are presented in Supplementary Table S1. The affinity tag was incorporated to facilitate the purification of insulin-CBT1 [29].

### 2.3. Purification of Insulin-CBT1 Protein from E. coli

The recombinant plasmid (pET22b::insulin-CBT1) was transformed into *E. coli* strain BL21 (Novagen, Madison, WI, USA), which was cultured in LB medium until the optical density at 600 nm reached 0.6. Overexpression of the insulin-CBT1 protein was initiated by the addition of 1 mM isopropyl-1-thio-β-D-galactopyranoside (IPTG) overnight at 30 °C. Cells were harvested and resuspended in a lysis buffer (Millipore, Billerica, MA, USA; 70584-4) containing benzonase. Next, bacterial cells were lysed by sonication, and inclusion bodies containing insulin-CBT1 protein were pelleted by centrifugation. Briefly, the pellet was washed twice with distilled water and dissolved in binding buffer (50 mM Tris-HCl, 7 M Urea, 0.15 M NaCl, 5 mM imidazole, and 5 mM 2-mercaptoethanol, pH 8.0). The solubilized inclusion bodies containing insulin-CBT1 were purified according to the method of Singh et al. [31]. To remove cell debris, the re-solubilized fraction was filtered through a 0.2 μm membrane (Pall Laboratory, Westborough, MA, USA). To purify insulin-CBT1 from the solubilized fraction, the latter was applied directly to a HisTrap HP column (5 mL; GE Healthcare, Uppsala, Sweden) pre-equilibrated with a binding buffer. The trapped insulin-CBT1 proteins were washed five times with five column volumes (CV) of binding buffer. Refolding was performed using a linear 7–0 M urea gradient [in refolding buffer (50 mM Tris-HCl, 0.15 M NaCl, 5 mM imidazole, 5 mM 2-mercaptoethanol pH 8.0)] through 20 CV at a flow rate of 0.1 mL/min. After loading, the column was washed five times with five CVs of binding buffer. Refolding of the bound protein was performed using a linear 7–0 M urea gradient in refolding buffer (20 CV at a flow rate of 0.1 mL/min). The refolded insulin-CBT1 was eluted with elution buffer (50 mM Tris-HCl, 0.15 M NaCl, 0.5 M imidazole, and 5 mM 2-mercaptoethanol, pH 8.0). The 2-mercaptoethanol and imidazole were removed through dialysis with PBS. For further purification, refolded insulin-CBT1 was loaded onto a HiLoad 26/600 Superdex 75 pg size-exclusion chromatography (SEC) column pre-equilibrated with PBS. Fractions were examined by visualization of insulin-CBT1 after separation in 4–12% gradient sodium dodecyl sulfate-polyacrylamide gel electrophoresis (SDS-PAGE) gels. Protein concentration was determined using the Bradford assay method, with bovine serum albumin as the standard. To identify insulin-CBT1 through mass analysis of N-terminal amino acids, insulin-CBT1 was subjected to N-terminal amino acid sequencing using Matrix Assisted Laser Desorption Ionization Time of Flight (MALDI-TOF) mass spectrometry analysis at the PROTEINWORKS Inc. (Daejeon, Korea).

### 2.4. Cell Proliferation and Damage Recovery Assays

Pancreatic cells (MIN6) were seeded in 96-well plates at a density of 1 × 10^3^ cells per well and incubated at 37 °C. After 24 h, c-insulin (10 nM) and insulin-CBT1 (10 nM) were added to each well for a further 48 h. Cell viability was determined using Cell Counting Kit-8 (Dojindo Laboratories, Tokyo, Japan), according to the manufacturer’s protocol. To investigate whether insulin analogs regenerate damaged 3T3-L1 cells damaged by streptozotocin (STZ), 3T3-L1 cells were seeded in 96-well plates at a density of 5 × 10^3^ cells per well and incubated at 37 °C. After 24 h, cells were exposed to 0.2 or 1.5 mM STZ for 1 h. Next, the medium was replaced with a fresh medium containing each insulin analog (10 nM). Next, cells were incubated for a further 48 h, and viability was measured using Cell Counting Kit-8 (Dojindo Laboratories, Tokyo, Japan). Absorbance was measured using a multifunctional microplate reader (SpectraMax M5; Molecular Devices, Sunnyvale, CA, USA).

### 2.5. Immunocytochemistry Using ImageXpress^®^ Micro Confocal Microscopy

MIN6 and 3T3-L1 cells were seeded onto coverslips in 6-well plates. After 24 h, 10 nM of each insulin analog was added to each well for a further 48 h. Cells were fixed for 15 min in 3% PFA at room temperature (RT) and then washed three times in PBS. For permeabilization, cells were incubated for 2 min with 0.2% Triton X-100 in PBS and then washed. To reduce background signals, cells were blocked for 30 min with 4% bovine serum albumin in PBS. Next, cells were incubated overnight at 4 °C with a rabbit monoclonal anti-His tag antibody, a rabbit monoclonal anti-insulin antibody, or a rabbit monoclonal anti-insulin receptor antibody (all from Cell Signaling Technology, Danvers, MA, USA) (see Supplementary Table S2). Relative expression and localization of target proteins were visualized using FITC-conjugated goat anti-rabbit IgG (Jackson ImmunoResearch) or Alexa Fluor 568-conjugated donkey anti-goat IgG (Invitrogen, Waltham, MA, USA) (see Supplementary Table S2). For nuclear staining, cells were incubated for 1 h at RT with 5 µg/mL Hoechst 33258 (Sigma, Burlington, MA, USA), rinsed three times in PBS, and mounted. To measure the Live/Dead ratio of MIN6 cells after treatment with insulin analogs, cells were visualized using a LIVE/DED^®^ Viability/Cytotoxicity Kit (Invitrogen). Images were obtained under an ImageXpress^®^Micro Confocal microscope (Molecular Devices) and then analyzed statistically using the in-built image analysis tools [32].

### 2.6. Western Blot Analysis

Soluble proteins isolated from MIN6 and 3T3-L1 cells were extracted in RIPA buffer (Thermo Fisher Scientific, Waltham, MA, USA) containing a protease inhibitor cocktail (Roche, Mannheim, Germany). Following centrifugation, the supernatant was passed through a 0.2 μm filter. Next, proteins (40 μg total) were separated by SDS-PAGE and transferred to a polyvinylidene difluoride (PVDF) membrane (Amersham Bioscience, Piscataway, NJ, USA). Blotted membranes were blocked in 5% skimmed milk diluted in T-TBS and incubated overnight at 4 °C with appropriate primary antibodies (Cell Signaling Technology); all antibodies were diluted 1:1000 (Supplementary Table S2). The membranes were washed three times (each for 15 min) with T-TBS and then blocked in 5% skimmed milk diluted in T-TBS. The membranes were then incubated for 1 h with HRP-linked secondary antibody (Cell Signaling Technology) overnight at 4 °C. Beta-actin was used as an internal control. Protein bands were detected using an enhanced chemiluminescence kit (Millipore, Billerica, MA, USA), followed by autoradiography using a Chemi-doc™ Touch Imaging System (Bio-Rad Laboratories, Hercules, CA, USA).

### 2.7. Chromatographic Characterization

Non-reducing reverse phase-high performance liquid chromatography (RP-HPLC) was performed on an Agilent 1290 HPLC System fitted with a ZORBAX 300SB-C8 column (Agilent, Santa Clara, CA, USA). Mobile phase A was water/0.1% TFA. Mobile phase B was acetonitrile/0.1% TFA. Chromatography was performed at a flow rate of 1.0 mL/min at 60 °C and monitored at UV 280 nm [33].

### 2.8. Fluorescence Quenching Analysis

Fluorescence measurements were obtained by monitoring changes in intrinsic fluorescence emission by c-insulin or insulin-CBT1 samples (0.1 mM) using a Gemini EM Microplate Reader (Molecular Devices, Sunnyvale, CA, USA) [34]. The quenching of tyrosine (Tyr) fluorescence emission spectra was recorded between 280 and 400 nm, following excitation at 295 nm. Each spectrum comprised an average of three acquisitions and was corrected for the contribution of the experimental solution without protein [35].

### 2.9. Circular Dichroism (CD)

Far UV CD spectra (190–260 nm) were recorded with a CD Detector. Each insulin sample was used for far ultraviolet (UV)–CD spectral analysis with a Circular Dichroism Detector (Applied Photophysics, Surrey, UK) as described previously [36,37].

### 2.10. Prediction of a 3D Model Using Protein Homology/Analogy Recognition Engine 2 (Phyre2)

Molecular modeling of insulin analogs was performed using protein homology/analogy recognition engine 2 (Phyre2) [38]. Phyre2 is a free online server for homology modeling that uses principles of homology modeling to generate reliable protein models, resulting in extensive improvements in the accuracy of three-dimensional (3D) structure/function and mutation prediction [39]. The Phyre2 server is available at http://www.sbg.bio.ic.ac.uk/phyre2, accessed on 21 August 2023.

### 2.11. LC-MS/MS Analysis and Database Searches

The analysis of disulfide bond formation was performed by PROTEINWORKS Inc. (Daejeon, Korea) using LC-MS/MS. Prior to analysis, the sample was digested with chymotrypsin and then divided into two. The first sample was reduced with 10 mM dithiothreitol (DTT), followed by alkylation with 20 mM iodoacetic acid (IAA). The second sample was prepared without any reduction and alkylation. Each sample was analyzed using a Q Exactive plus mass spectrometer (Thermo) equipped with a Vanquish Ultra-high-performance liquid chromatography column (Thermo). All MS/MS spectral data were analyzed manually for peptide identification. Oxidized methionine and carbamidomethylated Cys (only in the reduced and alkylated protein sample) were considered modifications [40].

### 2.12. Adipocyte Differentiation and Glucose Uptake by 3T3-L1 Cells in Response to Insulin Analogs

3T3-L1 cells were maintained in glucose supplemented DMEM medium containing 25 mM glucose, 10% FBS, and antibiotics (1% penicillin and 1% streptomycin) as a complete medium (CM). For adipocyte differentiation, pre-adipocytes were stimulated in CM supplemented with 10 µg/mL insulin, 0.5 mM Methyl-isobutyl-xanthine (IBMX), and 1 µM dexamethasone (pre-adipocytic CM). On Day 3 of pre-adipocyte differentiation, the adipogenic cocktail was changed to CM containing 10 µg/mL insulin (mature-adipocytic CM). The cells were cultivated in mature-adipocytic CM until Day 3 and then refed every 3 days according to the modified procedure described previously [41,42]. Images of lipid droplet green (Funakoshi, Tokyo, Japan)-stained adipocytic cells that had acquired the adipocyte morphology were obtained under an ImageXpress^®^Micro Confocal microscope (Molecular Devices) and analyzed statistically using its image analysis tools.

To determine the glucose uptake ratio of 3T3-L1 exposed to insulin analogs after initiation of differentiation, cells were exposed to a decreased glucose level and monitored using Accu-Chek Instant (Roche Diabetes Care GmbH, Mannheim, Germany) [43]. Furthermore, glucose absorption by 3T3-L1 cells, depending on the presence of insulin analogs, was measured using a colorimetric glucose uptake assay kit (Abcam, Cambridge, MA, USA) [44].

### 2.13. Construction of the Plasmid Harboring Insulin-CBT1 Gene for Use in the Pediococcus pentosaceus SL4 DDS

The ATUM-codon optimized DNA fragment of *insulin-CBT1* (ATUM. Bio) was cloned into the plasmid pCBT24-2 (KCCM12182P). The dual promoter system selected for maximum expression of *insulin-CBT1* was ligated to an usp45 secretion signal peptide, thereby enabling the synthesis of DNA fragments (Supplementary Table S1). A portion of each promoter ligated to the signal peptide was digested with NheI/SalI and BamHI/PstI restriction enzymes, respectively. DNA fragments encoding G6Pi-usp45-insulin-CBT1 were inserted at NheI/SalI and BamHI/PstI restriction enzyme sites of the pCBT24-2 expression vector. Finally, the pCBT24-2-G6Pi-usp45-insulin-CBT1-G6Pi-usp45-insulin-CBT1 plasmid was transformed into PP cells [29].

### 2.14. Transformation of Pediococcus pentosaceus SL4 and Detection of Insulin-CBT1 Proteins in the Culture Supernatant

Transformants grown on MRS agar plates were inoculated into 10 mL of MRS broth containing 10 mg/mL erythromycin and cultured at 37 °C for 15 h (no shaking). Next, 1 mL of pre-culture was inoculated into 10 mL of M9 minimal medium containing 10 mg/mL erythromycin and cultured at 37 °C for 48 h (no shaking). Next, 5 mL of culture was centrifuged, and the supernatant was collected. The supernatant was concentrated by Trichloroacetic acid (TCA) precipitation to isolate total protein. Finally, the insulin-CBT1 protein was detected by Western blotting [29].

## 3. Results and Discussion

### 3.1. Design Strategy for Single-Chain Insulin (Insulin-CBT1) Expressed in E. coli

Prokaryotic expression (in *E. coli*) of proinsulin is a relatively recent achievement; prior to this, the vast majority of insulin analogs were prepared by genetic engineering of the h-insulin gene [6,45]. Production of active r-insulin since the gene product of pre-proinsulin undergoes PTM steps before it becomes biologically active (Figure 1A). To produce recombinant insulin-CBT1, we designed a codon-optimized synthetic single-chain insulin-*CBT1* gene, and then *Insulin-CBT1* was ligated into pET22b and expressed as a fusion protein harboring a linker peptide (15 amino acids) instead of the C-peptide, in addition to a 6×His tag at the N-terminus (Figure 1B). This meant that the majority of the insulin-CBT1 protein was produced in inclusion bodies. After refolding, purification was performed through a two-step procedure consisting of affinity chromatography and SEC. The products of each purification step were visualized by SDS-PAGE (Supplementary Figure S1A). Additionally, we examined disulfide bond formation, which is a critical factor for the structural stability of insulin-CBT1 and INSR binding. For this, insulin-CBT1 was analyzed by SDS-PAGE under reducing and non-reducing conditions (Supplementary Figure S1B). The molecular weight (MW) of insulin-CBT1 estimated by LC-MS analysis was 8627.7 Da; the theoretical molecular mass was 8628.6 Da (Supplementary Figure S1C).

### 3.2. Biophysical Differences Between Insulin-CBT1 and c-Insulin

**Figure 2:**
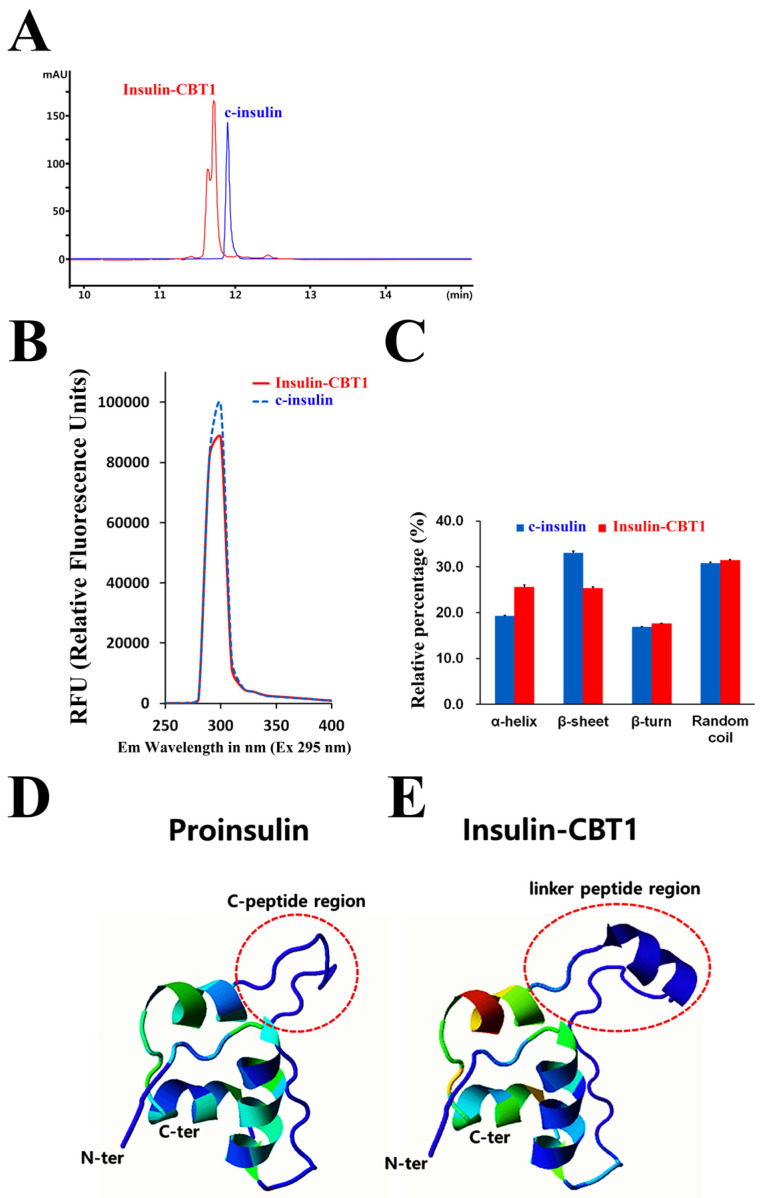
Biophysical changes in insulin-CBT1 analogs (compared with c-insulin). (**A**) To investigate biophysical changes in insulin-CBT1 caused by protein engineering, we examined changes in polarity using RP-HPLC. C-insulin was used as a template. (**B**) Intrinsic tyrosine fluorescence was used to investigate quenching caused by structural changes in insulin-CBT1. C-insulin was used as a template. Insulin-CBT1 contains five tyrosines (Tyr), whereas c-insulin contains only four. (**C**) To investigate changes in the secondary structure of insulin-CBT1 caused by protein engineering, we measured changes in the far UV–CD spectrum. Comparison of the secondary structure index values based on the far UV-CD spectra. In addition, to investigate structural differences between human proinsulin and insulin-CBT1, the three-dimensional structures of insulin analogs were predicted based on protein sequences using the Phyre2 program. The three-dimensional structure of proinsulin (**D**) was compared with that of insulin-CBT1 (**E**). The red circles indicate the C-peptide region of proinsulin or the linker peptide of insulin-CBT1.

To characterize the biophysical differences of insulin-CBT1, we compared it with mature c-insulin to examine the biophysical effects of protein engineering-mediated structural changes (protein engineering of h-insulin might alter its polarity and affect refolding, homo-oligomerization, or INSR binding). C-insulin eluted slightly later than insulin-CBT1 upon RP-chromatography, suggesting that insulin-CBT1 has increased polarity (Figure 2A). To investigate additional structural changes, we measured the intrinsic Tyr fluorescence of c-insulin (Tyr number = 4) and insulin-CBT1 (Try number = 5) to assess the degree of structural change. Protein engineering-mediated partial unfolding or changes to the local environment surrounding Tyr residues might alter the spectrum intensity (Figure 2B). Figure 2C shows the effect of biophysical changes related to the secondary structure of insulin-CBT1 (α-helix, β-sheet, β-turn, and random coils). Compared with intact c-insulin, we saw a significant difference in the secondary structure parameters of insulin-CBT1. There was a marked increase in α-helices, with a reduction in β-sheets. There were no differences with respect to β-turns and random coils. In addition, to characterize the biophysical and secondary structural differences between insulin-CBT1 and c-insulin, we used the Phyre2 program. The predicted structure of proinsulin and insulin-CBT1 consists of three- and four-helix bundles, respectively. The principal secondary structure of proinsulin and insulin-CBT1 is the α-helix. The B chain of proinsulin comprises one helix (Gly8-Cys19), whereas the A chain comprises two helices (Ile67-Thr73 and Leu78-Tyr85) (Figure 2D). In insulin-CBT1, the B chain comprises one helix (Gly23-Cys34), as does the linker peptide (Gly51-Gly57), whereas the A chain comprises two helices (Ile62-Thr68 and Leu73-Tyr79) (Figure 2E). This result exactly accords with the secondary structure change of insulin-CBT1 (Figure 2C).

### 3.3. Disulfide Bond Pairing in Insulin-CBT1

**Figure 3:**
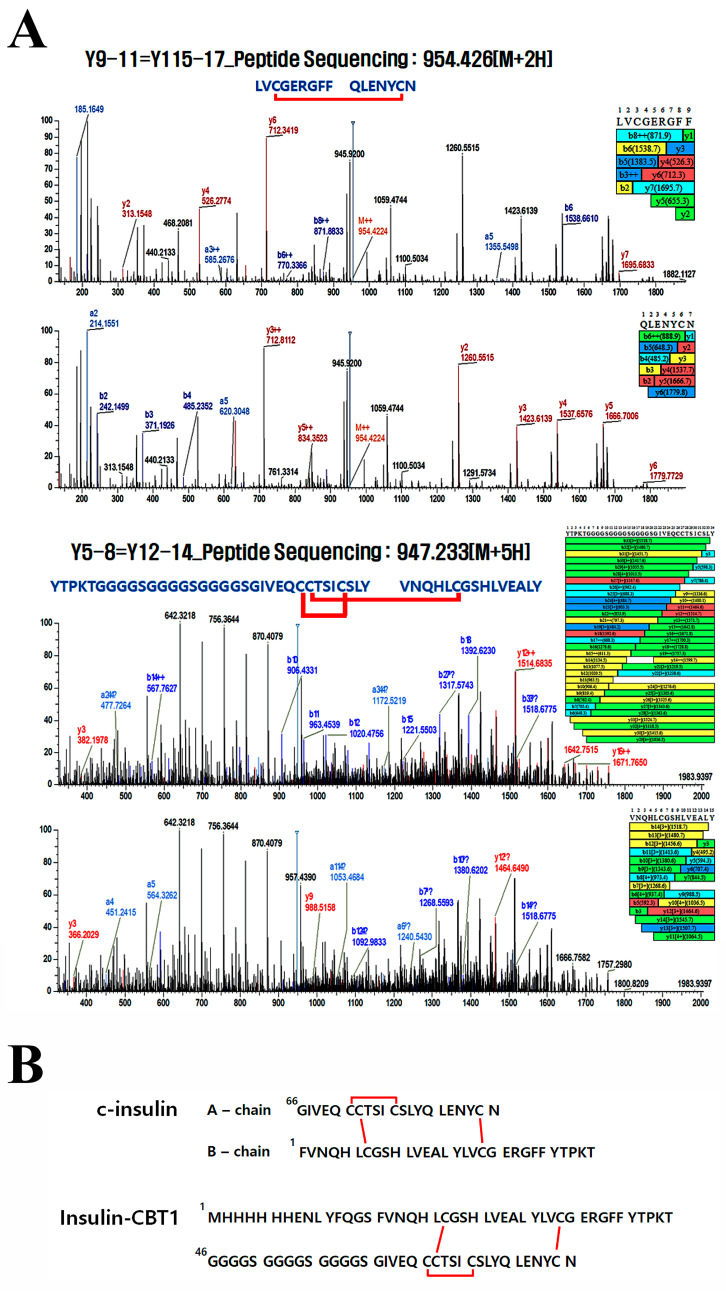
Disulfide bond pairing in insulin-CBT1. (**A**) To determine disulfide bond pairs in insulin-CBT1, the MS/MS spectra of inter- or intra-disulfide-linked dipeptides were analyzed using Q Exactive Plus. Manual assignment of MS/MS spectra revealed fragments of the peptide backbone. (**B**) Schematic illustration of inter- or intra-disulfide bonds in the insulin analogs.

Mature-formed insulin shows a globular protein comprising two polypeptide chains: A (21 residues) and B (30 residues). These chains are linked by two disulfide bonds (Cys7-Cys72 and Cys19-Cys85); the A chain also contains an intra-chain disulfide bond (Cys71-Cys76) [46]. Each of the three disulfide bonds is required for structural stability, receptor binding, and biological activity [47]. Therefore, we determined whether insulin-CBT1 harbors the three disulfide bond pairings at the same position as c-insulin under different redox conditions (oxidation or reduction). All three disulfide pairs were present in insulin-CBT1 under both redox conditions (Table 1). Figure 3A shows a chromatogram from LC-MS/MS. All three disulfide bonds in insulin-CBT1 are in the same position as those in c-insulin (Figure 3B). Thus, we knew that protein engineering did not affect disulfide bond formation; furthermore, we carefully suggest that its biological function may be maintained based on structural equivalence.

### 3.4. Biological Activity of Insulin-CBT1 in MIN6 Cells (Pancreatic Beta-Cells)

**Figure 4:**
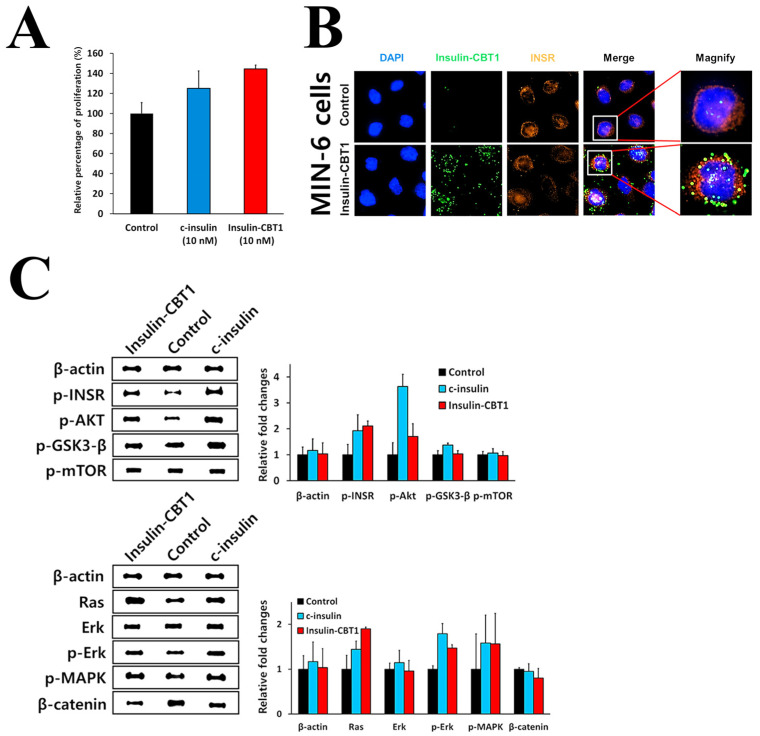
Biological effects of insulin-CBT1 in pancreatic β-cells. (**A**) To investigate whether exogenous recombinant insulin increases proliferation (%) of pancreatic β-cells (MIN6), cells (1 × 10^3^ cells/well) were treated with 10 nM of each insulin analog for 3 days. Serum-containing medium was used as a negative control. Proliferation was measured using a LIVE/DED^®^ Viability/Cytotoxicity Kit. Images were obtained under an ImageXpress^®^Micro Confocal microscope and analyzed using the in-built image analysis tools. (**B**) Co-location of insulin-CBT1 and INSR on the cell membrane was examined using ImageXpress^®^ Micro Confocal microscopy (mag. ×60). Green, Insulin-CBT1 protein; Red, INSR; Blue, DAPI-stained nucleus. (**C**) Effect of insulin analogs on the expression of proteins involved in the insulin pathway of MIN6 cells. Protein expression was examined by western blotting. The results presented are the average of three independent experiments, each with duplicate samples.

Insulin production by beta-cells is necessary to maintain normal blood glucose levels. Therefore, loss of insulin has catastrophic consequences. Recombinant insulin is a life-saving hormone for people suffering from type 1 and, sometimes, type 2 DM [46]. To investigate the improved properties of insulin-CBT1, we compared its basic biological properties with those of c-insulin. Studies propose a number of mechanisms to explain the proliferation of β-cells [48]; however, the effects of nano-molar doses of insulin analogs on β-cell replication are unclear. Figure 4A shows that 10 nM insulin-CBT1 stimulated β-cell replication by ~44%, whereas the same amount of c-insulin stimulated replication by only ~25%. If insulin-CBT1 can promote β-cell re-generation, it may help to overcome difficulties with DM therapy. Johnson et al. demonstrated that low doses of insulin protect β-cells from apoptosis because the β-cell mass is maintained through a balance between apoptosis and proliferation [49,50]. However, we did not observe the recovery of β-cells damaged by STZ [51] (Supplementary Figure S2). Although we observed the over-production of insulin in response to GLP-1 (positive control) (Supplementary Figure S3B), dose-dependent feedback inhibition of insulin production by insulin-CBT1 in β-cells was stronger than that elicited by the same concentrations of c-insulin [52] (Supplementary Figure S3A). To confirm that insulin-CBT1 targets β-cells (MIN6), we examined the co-localization of insulin-CBT1 with the INSR using an ImageXpress^®^Micro Confocal microscope (Molecular Devices) (Figure 4B).

Insulin is a major coordinator of important metabolic functions and mitogenic effects [53]. After insulin binds to INSR, insulin mediates phosphorylation through a complex network to activate glucose metabolic- or mitogenic signaling pathways. Here, we investigated mitogenic signaling pathways in MIN6 cells: insulin → INSR → Ras → ERK → MAPK; insulin → INSR → AKT → NF-κB; and insulin → INSR → GSK3-β → β-catenin (Figure 4C). The results suggest that insulin-CBT1 elicits mitogenic effects that are commonly linked to activation of both MAPKs and AKT cascades, leading to INSR-mediated cell proliferation and survival. These data suggest that insulin-CBT1 affects MIN6 cells in the same way as c-insulin.

### 3.5. Biological Activity of Insulin-CBT1 in 3T3-L1 Cells (Pre-Adipocytic Cells)

Insulin-induced adipocyte differentiation plays a primary role in energy homeostasis via the synthesis and storage of triglycerides in the form of free fatty acids and glycerol [54]. To trigger adipocyte differentiation, insulin stimulates glucose uptake in peripheral tissues to control glucose homeostasis [55].

**Figure 5:**
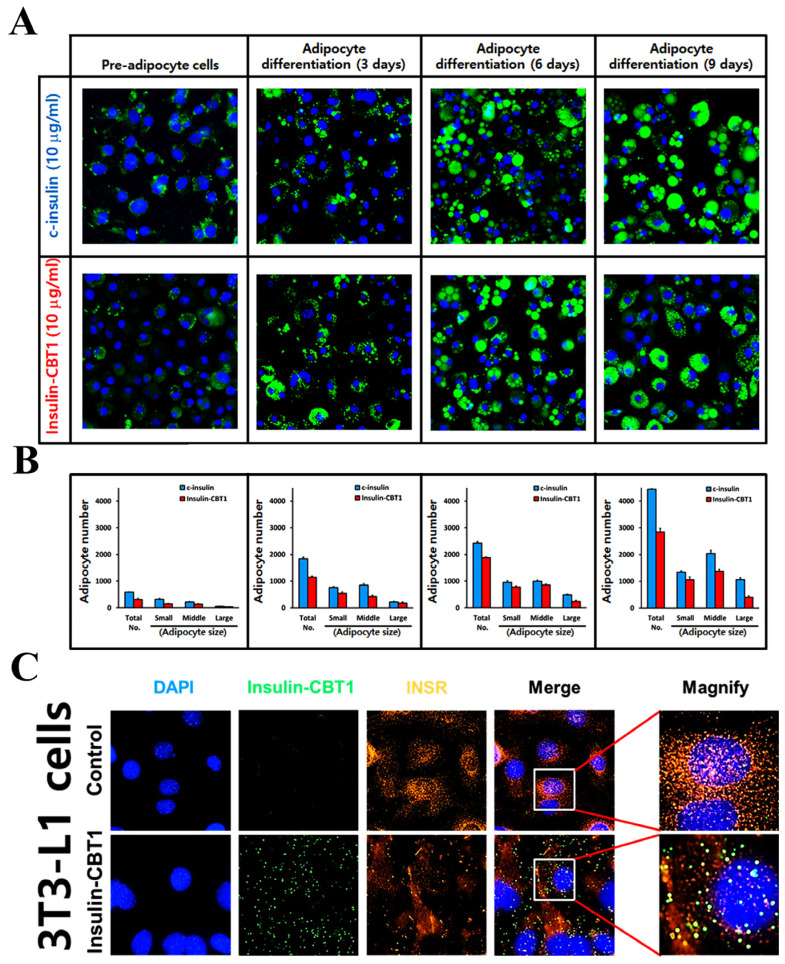
Adipocytic effects and INSR binding of insulin-CBT1 in pre-adipocyte cell (3T3-L1). (**A**) To investigate whether exogenous insulin-CBT1 drives adipocyte differentiation in 3T3-L1 cells, cells were treated with 10 nM of each insulin analog for 9 days. Images of lipid droplet green-stained adipocytic cells obtained under an ImageXpress^®^Micro Confocal microscope (magnification, ×20) reveal adipocyte morphology. (**B**) The diameter of adipocytes was analyzed using the image analysis tools in the microscope software (MetaXpress® Software). Adipocytes are classified depending on their size: Large (≥3 μm), Middle (1.5–3 μm), and Small (≤1.5 μm). (**C**) Co-localization of insulin-CBT1 and INSR on the cell membrane was examined using ImageXpress^®^ Micro Confocal microscopy (magnification, ×60). Green, Insulin-CBT1 protein; Red, INSR; Blue, DAPI-stained nucleus.

To compare insulin-CBT1-induced adipocyte differentiation with that of c-insulin, we differentiated 3T3-L1 pre-adipocytes into adipocytes. We observed adipocyte differentiation under an ImageXpress^®^Micro Confocal microscope (Molecular Devices) and obtained fluorescence images every 3 days during differentiation. At various time points, cells were stained with lipid droplet green (Funakoshi) to determine the degree of adipocyte differentiation (Figure 5A). As shown in Figure 5B, there was a clear time-dependent and significant accumulation of lipid droplets as cells differentiated; however, the efficacy of insulin-CBT1 was about ~39% less than that of c-insulin. To confirm that insulin-CBT1 targets pre-adipocytes (3T3-L1), we examined co-localization of insulin-CBT1 with the INSR using an ImageXpress®Micro Confocal microscope (Molecular Devices) (Figure 5C). 

**Figure 6:**
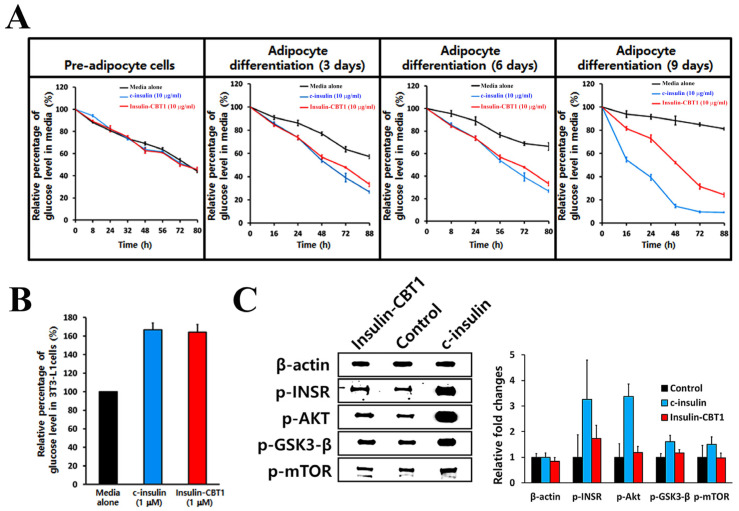
Glucose uptake activity and cell signaling of insulin-CBT1 in pre-adipocyte cell (3T3-L1). (**A**) To investigate whether exogenous insulin-CBT1 stimulates long-term glucose uptake by 3T3-L1 cells, cells were treated with 10 nM of each insulin analog for 9 days. Glucose uptake rate (%) by differentiating adipocytes in a medium containing a decreased amount of glucose (25 mM) was monitored using Accu-Chek Instant. (**B**) Short-term glucose absorption (%) by 3T3-L1 cells in the presence of each insulin analog was measured using a colorimetric glucose uptake assay kit (ab136955). (**C**) Effect of insulin analogs on the expression of proteins involved in the insulin pathway of 3T3-L1 cells. Protein expression was determined by Western blotting. The results presented are the average of three independent experiments, each with duplicate samples.

Furthermore, we compared the glucose uptake and absorption in the presence of insulin-CBT1 and c-insulin. Glucose uptake efficacy over the long term (3 days) was examined using Accu-Chek Instant (Roche, Boston, MA, USA, Diabetes Care GmbH). As shown in Figure 6A, glucose levels in the medium decreased in a time-dependent manner as cells differentiated; however, the efficacy of insulin-CBT1 was about 15% less than that of c-insulin.

In addition, we checked glucose absorption over a shorter time period. To determine glucose [2-deoxy-D-glucose (2-DG)] absorption by 3T3-L1 adipocytes in the presence of insulin-CBT1, we measured 2-DG levels in cells incubated with 2-DG for 20 min using a colorimetric glucose uptake assay kit (Abcam). As shown in Figure 6B, treatment of both insulin analogs immediately increased 2-DG levels in 3T3-L1 adipocytes, although the long-term glucose uptake efficacy of insulin-CBT1 was ~15% less than that of c-insulin.

Next, to verify the insulin-CBT1-derived adipocyte differentiation of 3T3-L1 cells, we investigated glucose metabolic signal transduction (insulin → p-INSR → p-AKT → pGSK-3β → p-mTOR) (Figure 6C). The results suggest that insulin-CBT1 may activate glucose metabolic signal pathways via activation of AKT to contribute to INSR-mediated glucose uptake and adipocyte differentiation. Taken together, the data suggest that insulin-CBT1 acts on 3T3-L1 cells in a manner similar to c-insulin.

### 3.6. Selection of Pediococcus pentosaceus SL4 Transformant and Detection of Insulin-CBT1 in the Culture Supernatant

**Figure 7:**
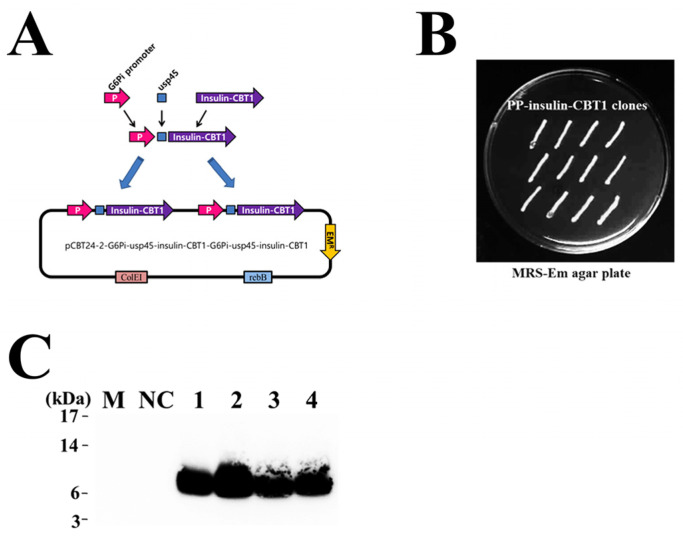
Development for the probiotics-based insulin-CBT1 delivery system. (**A**) Schematic showing the expression vector for PP-insulin-CBT1. (**B**) Selection of PP-insulin-CBT1 using MRS-Em agar plate. (**C**) The total insulin-CBT1 secreted into the culture supernatant (2 mL) was assessed by western blotting. Each total protein was harvested using the TCA precipitation method. M; protein size marker, NC; culture supernatant of PP-empty vector, 1–4; culture supernatant of PP-insulin-CBT1 clones.

To produce recombinant insulin-CBT1 in *P. pentosaceus*, we designed a codon-optimized synthetic insulin-CBT1 gene. Additionally, to increase insulin-CBT1 expression level in *P. pentosaceus*, we generated that DNA fragments encoding G6Pi-usp45-insulin-CBT1 were inserted at two different positions (NheI/SalI and BamHI/PstI) of the pCBT24-2 expression vector (Figure 7A). pCBT24-2-G6Pi-usp45-insulin-CBT1-G6Pi-usp45-insulin-CBT1 plasmid was transformed into *P. pentosaceus* cells, and then the transformant was selected by erythromycin (Figure 7B). The expression and secretion of insulin-CBT1 were evaluated using a Western blot (Figure 7C).

## 4. Discussion

Patients with DM (type 1 and type 2) survive by injecting insulin [2]; to keep up with increasing demand, insulin is now manufactured using diverse recombinant methods [56,57,58]. Insulin engineering offers three new routes for the construction of fast-acting insulin analogs, slow-acting insulin analogs, or high-affinity INSR agonists. To create a fast-acting insulin analog, protein engineering prevents oligomerization [59]. To achieve this, side chains with negative charges are created, which decreases specific contacts between dimers [60]. For slow-acting insulin analogs, protein engineering stabilizes either the insulin hexamer or forms crystals [61]. The increased stability of the hexameric structure slows down dissolution or delays absorption of the insulin solution from the subcutaneous layer, either by decreasing dissociation of the hexamer or via the formation of insulin microcrystals at the site of injection [60]. To create a high-affinity INSR agonist, several studies have examined how specificity and selectivity are conferred upon hybrid insulin. Hybrid insulin, which also contains insulin-like growth factor I (IGF-I), interacts strongly with the INSR; this is because the difference in the affinity of insulin and IGF-I for the receptor results from different residues interacting with various specificity-conferring regions on the INSR [25]. Finally, SCIs comprise a single polypeptide chain (B and A) linked via an uncleaved connecting peptide. These SCIs show low affinity for the INSR because the direct linkage of the insulin B and A chains leads to low flexibility that inhibits reverse turn folding [62]. Many studies have used linker peptides that form a flexible reverse-turn structure in SCIs [63]. Three-dimensional structural analysis based on correct disulfide bond pairing shows that these SCIs activate insulin receptors without the need for proteolytic cleavage [56].

Biological cleavage of the connecting C-peptide from proinsulin is required to generate active insulin because proinsulin has a lower affinity for the INSR than active insulin [24]. The insulin C-peptide may prevent the flexibility required for interaction with the INSR [57]. Rajpal et al. investigated the effect of various length linker peptides on the bio-activity of SCIs; they showed that the linker affects flexibility, which determines the three-dimensional structure, including disulfide bond pairing and surface net charge [64]. Here, we generated the single-chain insulin for commercial production in *E.coli*. Although prokaryotic expression systems have been used for insulin production, prokaryotic cells lack essential cellular machinery or endogenous enzymes required for the correct folding and processing of proinsulin to insulin; therefore, most recombinant SCIs are formed in inclusion bodies in bacterial cells.

Insulin-CBT1 required refolding and isolation from inclusion bodies to obtain a soluble active form (Supplementary Figure S1); this is because the biological activity of insulin depends critically on its structure. Therefore, we examined disulfide bond pairing in insulin-CBT1 using LC-MS/MS (Table 1 and Figure 3). This confirmed disulfide bond pairs between the same amino acids and at the same positions as in c-insulin. We then compared the three-dimensional structure of proinsulin and insulin-CBT1 using Phyre2 prediction software (version 2.2) (Figure 2D,E). The position of the C-peptide region in proinsulin overlapped with the linker peptide of insulin-CBT1; also, a new α-helix region in the linker peptide of insulin-CBT1 increased the α-helix content observed by CD-spectral analysis (Figure 2C). This might suggest that the structural position of the linker peptide region might have a negative effect on the biological activity of insulin; indeed, Peavy et al. demonstrated that the C-peptide of proinsulin prevents INSR binding [24]. Thus, we examined the biological activity of insulin-CBT1 in MIN6 and 3T3-L1 cells. We examined the ability of insulin-CBT1 to induce proliferation and insulin production by MIN6 cells; the results showed that c-insulin increased proliferation by up to 25% and insulin-CBT1 stimulated proliferation by up to 44% (Figure 4A). Both insulin analogs showed negative effects on insulin expression via feedback inhibition, as reported by Draznin et al. (Supplementary Figure S3A) [53]. However, recombinant GLP-1 increased endogenous insulin production by MIN6 cells by up to ~40% (Supplementary Figure S3B). Insulin analogs were unable to rescue insulin production by cells damaged by STZ (Supplementary Figure S3A). We also observed that insulin-CBT1 co-localization with the INSR on the membrane of MIN6 cells mediated phosphorylation of the AKT or ERK pathways (Figure 4C). Thus, insulin-CBT1 is a stronger stimulator of MIN6 cells than c-insulin, although it shares structural similarities with proinsulin (an inactive form of insulin).

Furthermore, we examined the effect of insulin-CBT1 on differentiation and glucose uptake by 3T3-L1 cells. Adipocyte differentiation triggered by insulin CBT1 was ~61% of that triggered by c-insulin (Figure 5A,B), whereas long-term glucose uptake was ~85% of that triggered by c-insulin (initial glucose uptake triggered by c-insulin was significantly higher than that by insulin-CBT, resulting in an earlier saturation point at 48 h) (Figure 6A). To determine short-term glucose uptake triggered by insulin-CBT1, we measured 2-DG in 3T3-L1 cells for 20 min; both insulin analogs showed similar results (Figure 6B). We observed co-localization of insulin-CBT1 with the INSR on the membrane of 3T3-L1 cells and found that insulin-CBT1 mediated phosphorylation of the AKT-GSK3β-mTOR pathway (Figure 6C).

Taken together, the results of the present study demonstrate that it is possible to generate recombinant insulin-CBT1 capable of binding to the INSR on MIN6 and 3T3-L1 cells, even though its structure is very similar to that of proinsulin containing the C-peptide. Furthermore, insulin-CBT1 effectively stimulates the proliferation of MIN6 cells, although it cannot rescue insulin secretion after STZ-induced damage. Most importantly, insulin-CBT1 can increase the proliferation of pancreatic beta-cells if they have not suffered severe damage. Also, insulin-CBT1 stimulates both glucose uptake and adipocyte differentiation in pre-adipocytic without the need for post-translational processing. Taken together, insulin-CBT1 is able to produce bacteria without the need for proteolytic enzyme digestion and re-purification.

The first attempt was made for oral insulin treatment in 1992 by presenting various challenges for developing and overcoming the barriers to insulin stability in the digestive tract [65]. To increase insulin stability in the gastrointestinal tract when it undergoes ingestion. We developed a therapeutic supporting approach, *P. pentosaceus* SL4-based DDS, by installing an insulin-CBT1 secretion system for DM therapy. This approach is by which insulin-CBT1 could be delivered orally to patients. Genetically modified *P. pentosaceus* cells are located on the inner surface of the intestine after ingestion; therefore, insulin-CBT1 would have to pass through the intestine wall, translocating it to the spread host body. To carry out this approach, we first constructed pCBT24-2-G6Pi-usp45-insulin-CBT1-G6Pi-usp45-insulin-CBT1 plasmid for expression and secretion of insulin-CBT1 in *P. pentosaceus* (Figure 7A). We next isolated transformant (PP-insulin-CBT1), which clones are able to secrete insulin-CBT1 proteins in culture media (Figure 7B). In a further study, oral administration of insulin-CBT1 will be evaluated for its properties (insulin delivery and anti-DM activity) using the DM mouse model (high-fat diet model).

## 5. Conclusions

In conclusion, the results presented herein open up new avenues to developing therapeutic options for DM. Probiotic bacteria as living supplementary organisms are beneficial to the host; in addition, these bacteria are safe, with a history of use as a foodstuff going back thousands of years [66,67]. Although some of the safety concerns associated with genetic engineering need to be overcome, probiotics are recently considered as a bio-vehicle for drug delivery [27].

Thus, we believe that insulin proteins could be delivered as a probiotic or via food products designed specifically for DM patients.

## Figures and Tables

**Table 1:** Disulfide bond pairing in insulin-CBT1.

Theoretical Mass (Y)					Practical Mass	
Fragment No.	Residue No.	S=S Bond	*m*/*z*	Charge	*m* /*z*	RT
Y9–11 = Y15– 17	32–40 = 75–81	Cys34=Cys80	636.620	3	636.619	25.43
Y12–14 = Y15–17	41–74 = 75–81	Cys66=Cys71 or Cys67=Cys80	983.922	4	983.916	26.47
Y5–8 = Y12–14	17–31 = 41–74	Cys66=Cys71 or Cys67=Cys22	947.233	5	947.231	28.17

## Data Availability

All data are contained in this study.

## Supplementary Materials

The following supporting information can be downloaded at https://www.mdpi.com/article/10.3390/cimb47030137/s1.

## Abbreviations

SCI: Single-chain insulin; DM: Diabetes mellitus; c-insulin: Commercial insulin; INSRs: Insulin receptors; RP-HPLC: Reverse phase-high performance liquid chromatography; CD: Circular dichroism; PTM: Post-translational modification; LB broth: Luria–Bertani broth; IPTG: Isopropyl-1-thio-β-D-galactopyranoside; CV: Column volumes; SEC: Size-exclusion chromatography; SDS-PAGE: Sodium dodecyl sulfate-polyacrylamide gel electrophoresis; MALDI-TOF: Matrix-assisted laser desorption ionization-time of flight; STZ: Streptozotocin; RT: Room temperature; PVDF: Polyvinylidene difluoride; Tyr: Tyrosine; UV: Ultraviolet; Phyre2: protein homology/analogy recognition engine 2; DTT: Dithiothreitol; IAA: Iodoacetic acid; CM: Complete medium; IBMX: Methyl-isobutyl-xanthine.
