# Supplementary material for: Development for Probiotics Based Insulin Delivery System
Source: Curr Issues Mol Biol. 2025 Feb 21;47(3):137. doi: 10.3390/cimb47030137 (PMC11941388; doi:10.3390/cimb47030137)
Supplement: Supplementary file 1 [file cimb-47-00137-s001.zip › supplementary table S1.pdf]

Supplementary table S1. Codon optimization for expression in *P. pentosaceus* SL4.

DNA	DNA Sequences
**Original**	TTTGTGAACCAACACCTGTGCGGCTCACACCTGGTGGAAGCT<br>CTCTACCTAGTGTGCGGGGAACGAGGCTTCTTCTACACACCCA<br>AGACCGGTGGCGGCGGATCTGGTGGTGGCGGATCGGGTGGGG<br>GCGGGTCAGGCATTGTGGAACAATGCTGTACCAGCATCTGCT<br>CCCTCTACCAGCTGGAGAACTACTGCAAC
**Codon optimized**	TTTGTGAATCAGCACTTGTGTGGTTCACATTTGGTTGAAGCTCT<br>TTACTTAGTTTGTGGTGAGCGTGGATTTTTCTATACCCCAAAAA<br>CGGGTGGCGGCGGATCTGGTGGTGGCGGATCGGGTGGGGGCG<br>GGTCAGGTATCGTAGAACAATGCTGTACTAGCATTTGCTCCTTA<br>TATCAATTAGAAAACTATTGTAAT
