# Supplementary material for: Development for Probiotics Based Insulin Delivery System
Source: Curr Issues Mol Biol. 2025 Feb 21;47(3):137. doi: 10.3390/cimb47030137 (PMC11941388; doi:10.3390/cimb47030137)
Supplement: Supplementary file 1 [file cimb-47-00137-s001.zip › supplementary table S2.pdf]

Supplementary table S2.

No.	Antibody name	Host	Cat. #
1	β-actin	Mouse	CS-47778
2	Insulin receptor (INSR)-β	Rabbit	CST 3025T
3	P-INSR	Rabbit	CST 3021S
4	P-AKT	Rabbit	CST 9271S
5	PGSK3-β	Rabbit	CST 9322S
6	P-mTOR	Rabbit	CST 5536T
7	Ras	Mouse	#415700
8	p38 MAPK	Rabbit	CST 9212S
9	P-p38 MAPK	Rabbit	CST 9211S
10	ERK (1/2)	Rabbit	CST 9102S
11	P-ERK (1/2)	Rabbit	CST 9101S
12	β-Catenin	Rabbit	CST 8814S
13	Insulin	Rabbit	ab181547
14	His-tag	Rabbit	ab14923
15	Insulin receptor (INSR)-α	Mouse	AHR0221
