# Supplementary figures and images for: Development for Probiotics Based Insulin Delivery System
Source: Curr Issues Mol Biol. 2025 Feb 21;47(3):137. doi: 10.3390/cimb47030137 (PMC11941388; doi:10.3390/cimb47030137)

## Development for probiotics based insulin delivery system

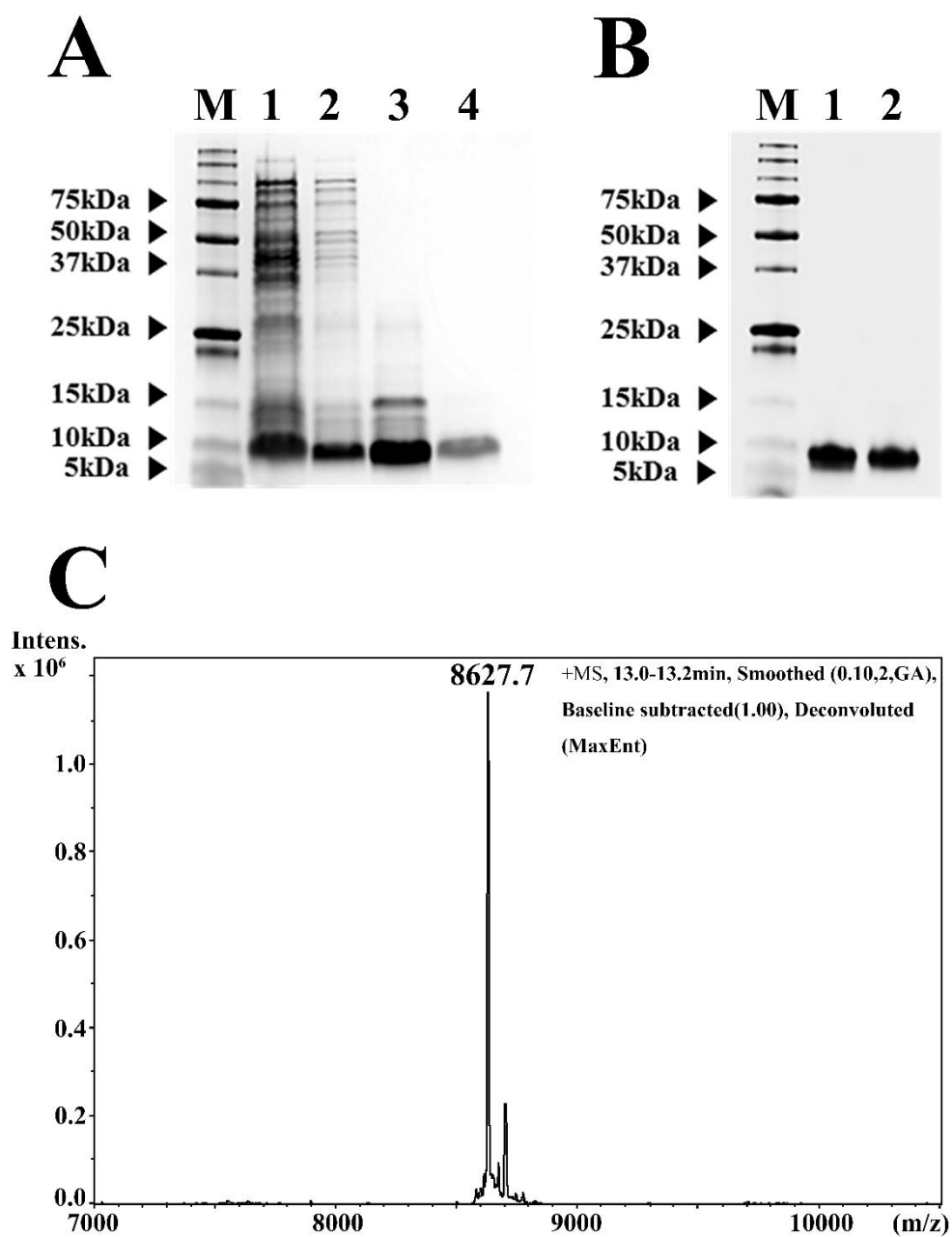

Figure S1

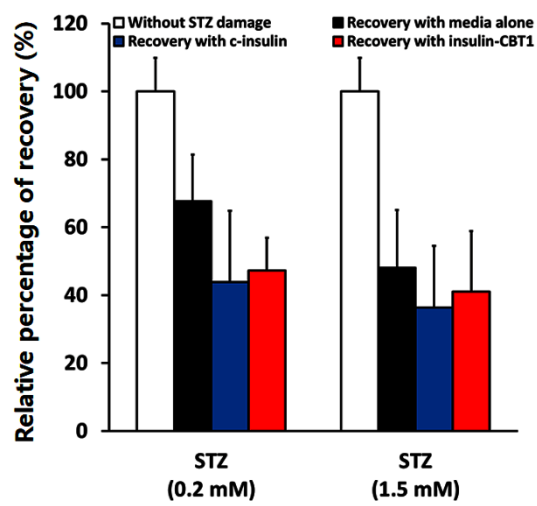

Figure S2

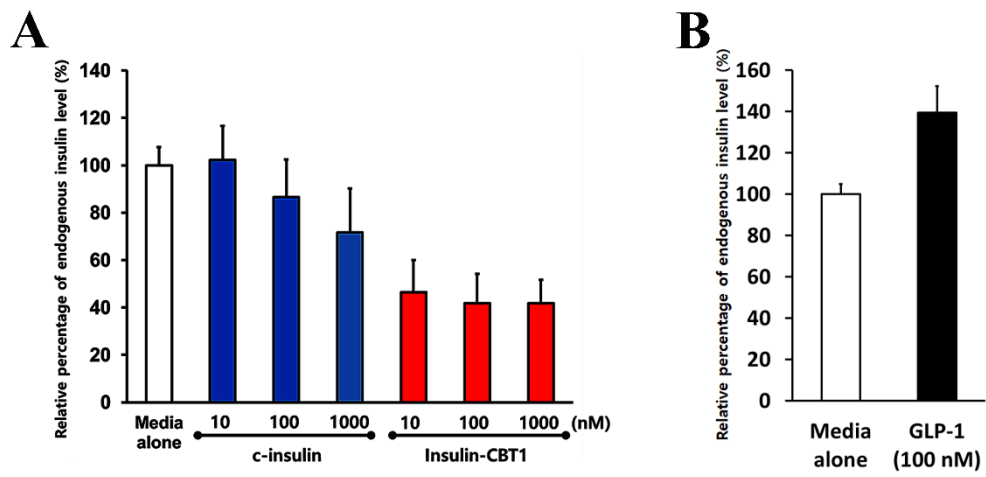

Figure S3

Supplement: Supplementary file 1 [file cimb-47-00137-s001.zip › cimb-3393253-supplementary-figures.pdf]
